# Vitamin D Receptor–Macrophage–IL-23 Axis in Inflammatory Bowel Disease: Pathogenic Mechanisms and Therapeutic Hope

**DOI:** 10.3390/jcm15114296

**Published:** 2026-06-02

**Authors:** Alexandra Grigoraș, Larisa Ghemiș, Ancuta Goriuc, Alice Murariu, Naomi Fiterman, Nura Jdid, Teofil Blaga, Georgeta Liliana Foia

**Affiliations:** 1 Grigore T. Popa University of Medicine and Pharmacy Iasi, 700115, Iasi, Romania; grigoras.alexandra@email.umfiasi.ro (A.G.); ancuta.goriuc@umfiasi.ro (A.G.); alice.murariu@umfiasi.ro (A.M.); naomi.fiterman@d.umfiasi.ro (N.F.); sara.jdid@icloud.com (N.J.); teofil_blaga@umfiasi.ro (T.B.); georgeta.foia@umfiasi.ro (G.L.F.); 2 “Sf. Spiridon” County Clinical Emergency Hospital, 700111 Iasi, Romania

**Keywords:** inflammatory bowel disease, IL-23, vitamin D receptor, macrophages, inflammatory response

## Abstract

Inflammatory bowel disease (IBD), including Crohn’s disease and ulcerative colitis, is characterized by chronic intestinal inflammation driven by dysregulated immune responses in gut microbiota. Interleukin (IL)-23, a member of IL-12 cytokine family, has emerged as a key immune mediator, being produced mainly by macrophages from the intestinal mucosa. In recent years, vitamin D has become a pivotal immunomodulatory factor in IBD, vitamin D deficiency being commonly associated with this pathology. The immune effects of vitamin D are mediated through vitamin D receptor (VDR), widely expressed in macrophages and other immune cells. VDR signaling regulates pro-inflammatory macrophage activity and limits M1 polarization, therefore reducing IL-23 production and limiting Th17 driven inflammatory response. This review summarizes current evidence on the role of macrophage-derived IL-23 in IBD pathogenesis and highlights the modulatory effects of vitamin D/VDR signaling. In addition, it addresses the therapeutic relevance of targeting the VDR–macrophage–IL-23 axis in IBD.

## 1. Introduction

Inflammatory bowel disease (IBD), including Crohn’s disease and ulcerative colitis, represents a group of chronic inflammatory disorders of the gastrointestinal tract, characterized by dysregulated immune responses to intestinal microbiota [1]. Increasing evidence indicates that interleukin (IL)-23 is a central mediator of chronic intestinal inflammation and tissue damage, playing a pivotal role in the pathogenesis of IBD [2]. IL-23 is a member of the IL-12 cytokine family, largely produced by macrophages and other antigen-presenting cells in response to microbial stimulation [3]. Macrophages serve as major source of IL-23 in the intestinal mucosa and actively participate in the molecular crosstalk with T-cell subsets and innate lymphoid cells, contributing to the initiation and maintenance of chronic inflammation [4].

Vitamin D, beyond its classical role in phospho-calcium homeostasis, became an important immunomodulatory factor in IBD [5]. Numerous studies have reported a high prevalence of vitamin D deficiency (VDD) in IBD patients, which correlates with significant condition severity and negative disease outcomes [6]. At the molecular level, the immunoregulatory effects of vitamin D are mediated through activation of the vitamin D receptor (VDR), which is broadly expressed in macrophages and other immune cells [7]. VDR signaling regulates macrophage activation by pro-inflammatory pathways mitigation, restricting M1 polarization, and decreasing the production of IL-23 and IL-12, indirectly limiting T helper (Th) 17 and Th1 driven inflammatory responses [8].

Targeting the IL-12/IL-23 pathway has validated significant clinical benefit in immune-mediated inflammatory diseases. Ustekinumab, which blocks the shared IL-12/IL-23 p40 subunit, proved to be safe and effective in inducing and maintaining remission, with subsequent inflammation cutback and mucosal healing upgrade in IBD. More recently, newer therapeutic options, including Janus kinase (JAK) inhibitors such as upadacitinib and selective IL-23 p19 monoclonal antibodies such as risankizumab, have emerged, particularly for patients with prior biologic failure [9,10,11]. Adjunctive approaches, including vitamin D supplementation in patients with VDD, may also support standard IBD therapy, although current evidence remains insufficient [12].

This narrative review aims to summarize current evidence on the role of macrophage-derived IL-23 in IBD pathogenesis, with a particular focus on the modulatory effects of vitamin D and VDR signaling on macrophage function and downstream inflammatory cascades. In addition, we discuss and consider the therapeutic implications of targeting the VDR–macrophage–IL-23 axis, including the potential role of vitamin D supplementation and selective VDR agonists as adjunctive strategies in IBD management.

## 2. Overview of Inflammatory Bowel Disease (IBD)

IBD is defined as a relapsing and remitting condition characterized by chronic inflammation at different sites in the gastrointestinal tract. The two main categories of IBD, Crohn’s disease (CD) and ulcerative colitis (UC), differ in both, the location of lesions and the depth of intestinal wall inflammation [1,13]. UC predominantly affects the colonic mucosa, while CD can involve any part of the gastrointestinal tract, from mouth to anus, with transmural inflammation, often leading to fistulae or abscess formation. Conditions that cannot be distinctly categorized as either CD or UC are usually labeled as indeterminate IBD [14,15].

The increasing incidence of IBD in both Western and Eastern countries has positioned it as a major public health concern worldwide, making it one of the most common gastrointestinal tract conditions [16,17]. Moreover, the incidence of IBD is rising rapidly in newly industrialized countries, reflecting the growing impact of environmental and lifestyle factors [18]. This disease affects mainly young adults between 25 and 35 years of age, although approximately 20–25% of cases present early, during childhood and adolescence [19,20]. From a clinical perspective, patients affected by IBD experience not only gastrointestinal symptoms, such as abdominal pain, diarrhea, vomiting, nocturnal defecation and rectal bleeding, but also systemic issues including fatigue and depression. Depression is highly prevalent among individuals with IBD. In a systematic review and meta-analysis of studies assessing symptoms of anxiety and depression in patients with IBD, the pooled prevalence of depressive symptoms was 25.2%, increasing to 38.9% during active disease phases [1,21,22]. Beyond these symptoms, IBD can lead to extraintestinal complications, including ankylosing spondylitis, enteropathic arthritis, anemia, primary sclerosing cholangitis and inflammatory skin and eye disorders. Patients present also an increased risk of malignancies, particularly colorectal cancer and cholangiocarcinoma in UC, and rectal cancer in perianal or rectal CD [23]. Intestinal fibrosis represents one of the most serious long-term complications of IBD, particularly in CD, where it is associated with fibrotic strictures and stenotic to penetrating lesions, while being less common in the course of UC [24]. This pathological remodeling can lead to severe clinical consequences, including the formation of strictures and obstructions, as well as the development of fistulas in a substantial proportion of patients, particularly in CD, thereby contributing to persistent diarrhea and malabsorption [25].

The etiology of IBD is not completely clarified. Genetic factors, such as nucleotide-binding oligomerization protein 2 (NOD2) gene mutation, gut microbiota, immune response and environmental influences play critical roles in the pathogenesis of IBD [26]. Among the genetic factors, the IBD2 gene on chromosome 12, associated with UC, and the NOD2 gene on chromosome 16, linked to CD, are the best characterized [27]. IBD are driven by a dysregulated immune response to the gut microbiota, characterized by excessive activation of T helper cells, particularly Th1 and Th17, which promotes chronic inflammation and tissue damage through sustained production of pro-inflammatory cytokines [28]. Among the utmost cytokines involved in IBD pathogenesis are IL-23, Tumor Necrosis Factor α (TNF-α) and IL-6. In CD, chronic inflammation is driven by Th1 and Th17 cells, with Th1-related pro-inflammatory cytokines including Interferon-γ (IFN-γ), TNF-α, IL-6, and IL-12, and Th17-related cytokines including IL-17, IL-21, and IL-22. In contrast, UC is more commonly associated with Th2 and Th17 immune responses, involving Th2-related cytokines including IL-4, IL-5, IL-6, IL-13, IL-15, IL-33, and TNF-α, as well as Th17-related cytokines including IL-17, IL-21, and IL-22 [29].

**Figure 1:**
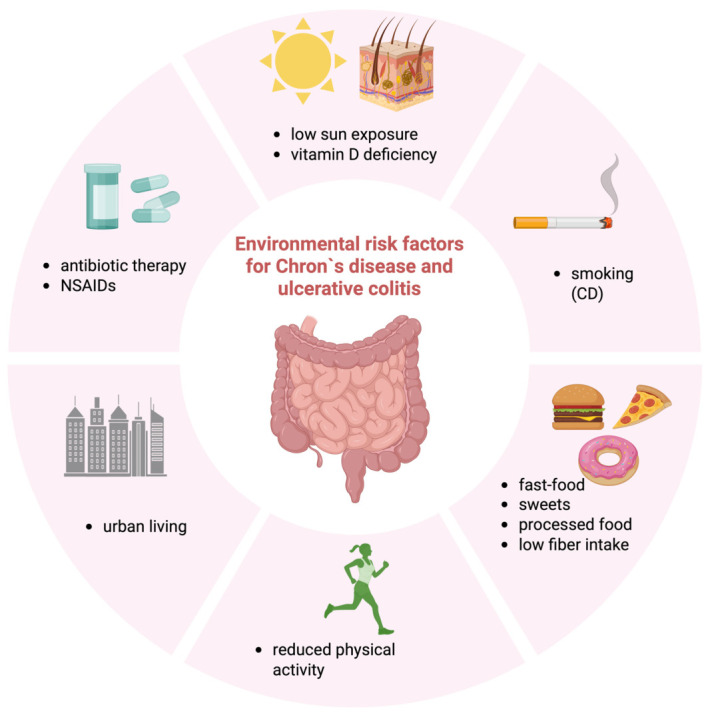
Environmental risk factors for inflammatory bowel disease (IBD). Created in BioRender. Grigoraș, A. (2026) https://BioRender.com/pyhanrh (accessed on 1 May 2026).

Environmental factors play a significant role in the development and progression of IBD, interacting with genetic susceptibility to influence disease onset and course (Figure 1). Urban living has been associated with increased IBD incidence, possibly reflecting reduced microbial exposure and lifestyle-related changes. Pharmacological exposures, particularly repeated antibiotic therapy and nonsteroidal anti-inflammatory drugs (NSAIDs), may disrupt microbial homeostasis and impair mucosal defenses [30]. Dietary habits characterized by high intake of red meat, fast food, refined sugars and processed products, combined with low fiber consumption and sedentary lifestyle or reduced physical activity, have been linked to enhanced intestinal permeability and pro-inflammatory immune activation [31]. Smoking exerts a differential effect, increasing the risk and severity of CD while appearing to have a protective effect in UC. This differential impact may be partially explained by nicotine, a major component of cigarettes, which has been shown to inhibit Th2 cell function without significantly affecting Th1 cell responses [32]. Additionally, VDD has emerged as an important environmental contributor, given its role in maintaining immune tolerance and controlling inflammatory responses [33]. In contrast, certain exposures are associated with a reduced risk of IBD, including breastfeeding, regular physical activity, early-life interactions with animals and diets rich in fruits, vegetables, green tea, and other plant-based components. These protective factors may support microbial diversity, strengthen barrier integrity and ultimately promote immune tolerance, thereby mitigating chronic intestinal inflammation [31].

## 3. The Role of IL-23 in IBD Pathogenesis

### 3.1. Molecular Structure and Signaling Pathways IL-23

The IL-12/IL-23 pathway plays a critical role in modulating mucosal immunity and in both the induction and maintenance of remission in chronic inflammation of the intestinal epithelium [34]. IL-12 is a heterodimeric cytokine composed of two subunits, p35 and p40. The IL-12 cytokine family comprises the heterodimeric cytokines IL-12, IL-23, IL-27, and IL-35. IL-23 shares the p40 subunit with IL-12 but contains a distinct p19 subunit instead of p35 [9]. IL-23, produced by tissue-resident myeloid cells in response to tissue injury or pathogenic stimuli, promotes the expansion and survival of Th17 cells, a subset of T lymphocytes that secrete IL-17A and other pro-inflammatory mediators, including IL-17F, IL-22, granulocyte–macrophage colony-stimulating factor (GM-CSF), IFN-γ, and TNF-α [2,35].

IL-12 and IL-23 receptors share the IL12Rβ1 subunit but differ in their second receptor chain, with IL-12 signaling through IL12Rβ2 and IL-23 through IL23R [36]. The p19 subunit of the IL-23 ligand establishes a critical interaction with the N-terminal immunoglobulin domain of IL-23R, triggering conformational changes in IL-23, while the p40 subunit simultaneously binds to IL-12Rβ1, thereby bridging the IL-23R and IL-12Rβ1 subunits [37]. Upon binding to its receptor, IL-23 activates the Janus kinase 2 (JAK2) and Tyrosine kinase 2 (Tyk2), which subsequently trigger Signal Transducer and Activator of Transcription 3 (STAT3) and STAT4 phosphorylation and their translocation to the nucleus, where they regulate target gene expression [36].

### 3.2. Macrophages as a Major Source of IL-23 in Chronic Gut Inflammation

Macrophages are innate immune cells that are primarily derived from circulating monocytes, although recent studies suggest that intestinal macrophages can also originate from resident macrophage populations. These cells play diverse roles, including cytokine secretion, clearance of cellular debris, and participation in inflammation, tissue repair, and angiogenesis [38]. They are widely distributed throughout the gastrointestinal mucosa, predominantly within the lamina propria adjacent to the epithelium, while a smaller population is located in the smooth muscle layer of the intestinal wall [39]. In response to microenvironmental signals, macrophages can polarize into either classically activated, pro-inflammatory M1 macrophages or alternatively activated, anti-inflammatory M2 macrophages, reflecting both antigenic and functional heterogeneity. M1 macrophages, driven by stimuli such as IFN-γ, LPS, or GM-CSF, are characterized by the production of pro-inflammatory mediators including inducible nitric oxide synthase (iNOS), TNF-α, IL-1, IL-2, IL-6, IL-12, IL-23 and reactive oxygen species (ROS), thereby contributing to the amplification of inflammatory responses. In contrast, M2 macrophages encompass multiple subtypes, including M2a, M2b, and M2c, induced by cytokines such as IL-4, IL-13, IL-10, TGF-β, or glucocorticoids, and function primarily to suppress immune activation, resolve inflammation, and promote tissue remodeling and repair. M1 macrophages promote Th1 responses, whereas M2 macrophages are associated with Th2 responses, secreting anti-inflammatory cytokines including IL-4, IL-10, IL-13 [8,40,41,42].

In the intestine, macrophages are regarded as a primary source of IL-23 and are thought to play a central role in molecular crosstalk with T-cell subsets and innate lymphoid cells within the gut. IL-23 is predominantly expressed by CD14+ intestinal macrophages, which play a central role in sustaining inflammation by infiltrating the inflamed intestinal tissue in CD patients [4]. Disruption of the epithelial barrier allows microbial invasion, activating M1 macrophages to overproduce inflammatory mediators, exacerbating tissue damage, impairing mucosal healing, and driving disease progression [40].

### 3.3. IL-23-Driven Th17 Responses in IBD

T cells are broadly categorized into pro-inflammatory and anti-inflammatory populations, comprising three principal subsets: CD8+ T cells, CD4+ T cells and regulatory T cells (Tregs). Cytotoxic CD8+ T cells mediate direct immune responses against infected and malignant cells, while CD4+ Th cells coordinate inflammation by shaping adaptive and innate immune responses. In contrast, Tregs act as key modulators of immune homeostasis, suppressing excessive inflammation and maintaining self-tolerance to prevent autoimmunity [43]. Naive CD4+ T cells can differentiate into Th1, Th2, induced regulatory T (iTreg), or Th17 subsets, under the influence of effector cytokines released by antigen-presenting cells (APCs), with Th17 cells, proposed to originate from a small subset of naive T cells expressing the lectin receptor CD161 [44].

A central immunopathological feature of IBD is the marked infiltration of the intestinal mucosa by inflammatory CD4+ T cells, particularly Th1 and Th17. Th1 cells are characterized by the expression of the transcription factor T-bet and the production of IFN-γ, whereas Th17 cells are defined by the expression of the transcription factor RORγt and the secretion of IL-17 [45]. RORγt is predominantly expressed in Th17 cells and plays a crucial role in their differentiation and development [46]. Th17 cells play a protective role by recruiting neutrophils and enhancing mucosal barrier defense against extracellular pathogens through the secretion of cytokines such as IL-17A, IL-17F, IL-22, and TNF-α. However, when Th17 responses are uncontrolled or dysregulated, they can trigger chronic inflammation and contribute to autoimmune diseases, including rheumatoid arthritis, multiple sclerosis, and systemic lupus erythematosus, through persistent IL-17-mediated tissue damage [47]. Th17 cells are elevated in both peripheral blood and intestinal mucosa during the active stage of IBD compared with the remission stage [48,49].

IL-23/IL-17 axis is particularly relevant in this context, as it plays a crucial protective role against bacterial and fungal infections. However, substantial evidence indicates that its impairment promotes chronic inflammation and autoimmunity, thereby contributing to the pathogenesis of several immune-mediated diseases, including IBD [50]. Some studies indicate that IL-23, predominantly secreted by innate myeloid cells, including activated dendritic cells, monocytes, and macrophages, is essential for the proliferation and maintenance of Th17 cells, although it does not promote commitment to an IL-17-secreting lineage [51,52]. Notably, mutations in the IL23R gene, which encodes a receptor subunit unique to IL-23, have been associated with IBD, psoriasis, and ankylosing spondylitis [51].

IL-17 exerts pro-inflammatory effects both independently and synergistically with TNF-α by acting on intestinal epithelial cells (IECs) to induce the production of inflammatory mediators, chemokines, and proteases, including IL-6, IL-8 (CXCL8), inducible nitric oxide synthase (iNOS), TNF-α, matrix metalloproteinases (MMPs), and GM-CSF. These factors collectively promote inflammation by recruiting, activating, and guiding neutrophils to target tissues, resulting in intestinal mucosal injury [44,48,49]. IL-17A is a member of the IL-17 cytokine family and functions as a potent pro-inflammatory mediator by expanding inflammation through the induction of TNF-α and IL-6, as well as neutrophil-associated genes such as CXCL1, CXCL2, and CXCL5 [53].

In CD, a subset of intestinal CD4+ T cells expresses high levels of natural Killer group 2 member D (NKG2D), and its co-stimulation with the TCR enhances their cytotoxic activity and drives the production of pro-inflammatory cytokines, including TNF-α, IFN-γ, and IL-17A. NKG2D serves as a more specific Th17 marker than CD161, with IL-17 production by CD4+ T cells strongly enhanced when both TCR and NKG2D are co-stimulated compared with TCR stimulation alone [54].

## 4. Vitamin D and Vitamin D Receptor in Intestinal Immune Regulation in IBD

### 4.1. Vitamin D Metabolism and VDR Signaling in the Intestinal Cells

Vitamin D is a fat-soluble secosteroid that occurs in two principal forms, vitamin D_2_ and vitamin D_3_. Vitamin D_2_ originates from plant and fungal sources, whereas vitamin D_3_ is synthesized in the skin through UVB-induced conversion of 7-dehydrocholesterol (7-DHC), although a proportion of vitamin D_3_ is also obtained from dietary sources, particularly animal-derived foods [55,56]. Vitamin D_2_ differs from vitamin D_3_ by the presence of an extra double bond between carbons 22 and 23 and a methyl group at carbon 24 in its side chain [57]. Vitamin D_3_ is transported to the liver, where it is hydroxylated by 25-hydroxylase (CYP2R1) to form 25-hydroxyvitamin D_3_ [25(OH)D_3_], the main circulating metabolite. Subsequently, 25(OH)D_3_ undergoes further hydroxylation in the kidneys by 1α-hydroxylase (CYP27B1) to generate the biologically active form, 1,25-dihydroxyvitamin D_3_ [1,25(OH)_2_D_3_] [55]. 1α-hydroxylase and 25-hydroxylase both belong to the cytochrome P450 mixed-function oxidase (CYP) enzyme family [58]. 25(OH)D_3_, characterized by a serum half-life exceeding 14 days and representing the predominant and most stable circulating vitamin D metabolite, is widely used as the standard biomarker for evaluating vitamin D status [59].

The VDR mediates the majority of the physiological effects exerted by 1,25(OH)_2_D_3_. Upon ligand binding, VDR heterodimerizes with the retinoid X receptor (RXR) in the nucleus. This VDR–RXR complex subsequently binds to vitamin D response elements (VDREs) located in the promoter regions of target genes, thereby initiating transcriptional activation. Among the genes regulated by VDR are those encoding antimicrobial peptides (AMP), including the cathelicidin precursor (LL-37) and β-defensin, highlighting the role of vitamin D signaling in immune defense and cellular homeostasis [60].

VDR expression is regulated in a cell-specific manner. For instance, 1,25(OH)_2_D_3_ modulates VDR in bone cells but not in the intestine. Besides 1,25(OH)_2_D_3_, factors such as growth factors, insulin, parathyroid hormone (PTH), glucocorticoids, estrogen, retinoic acid, and calcium can influence VDR levels, often through transcription factors including Activating Protein-1 (AP-1), Specificity protein 1 (SP1), CCAAT/Enhancer-Binding Protein (C/EBP), Caudal Type Homeobox 2 (CDX2), Runt-Related Transcription Factor 2 (Runx2), cAMP Response Element-Binding Protein (CREB), Retinoic Acid Receptor (RAR), and Glucocorticoid Receptor (GR). Conversely, Snail Family Transcriptional Repressor 1 and 2 (SNAIL1/2) repress VDR in certain cancer cell lines, and microRNAs like microRNA-125b (miR-125b), microRNA-298 (miR-298), and microRNA-27b (miR-27b) reduce VDR expression by binding its 3′ untranslated region [61].

Several polymorphisms of the VDR have been investigated for their impact on IBD, indicating that they may contribute to the variability in serum 25(OH)D_3_ levels observed in IBD patients [62]. Common VDR polymorphisms, such as ApaI, BsmI, FokI, and TaqI, have been extensively studied for IBD risk. Meta-analyses indicate that the ff genotype of FokI increases the risk of UC in Asian populations, whereas the ApaI “a” allele is protective against CD, and the TaqI tt genotype is linked to higher CD risk in Europeans. Additionally, the TaqI tt genotype is associated with a moderately increased risk of both UC and CD in men [63]. The VDR gene, located on chromosome 12, a region associated with IBD susceptibility, and the relatively reduced VDR activation reported in IBD patients compared with healthy individuals, underscore the importance of genetic variants in determining vitamin D status [62]. The vitamin D/VDR signaling pathway in intestinal epithelial cells is essential for preserving mucosal barrier integrity and maintaining immune homeostasis. In IBD patients, VDR expression is downregulated in the colonic mucosa and negatively correlates with disease activity and the severity of inflammation. Notably, VDR levels are reduced by approximately 50% in inflamed regions compared with non-inflamed areas of the colon [64].

### 4.2. Evidence of Altered Vitamin D Status in Patients with IBD

VDD is frequent in patients with IBD. A systematic review and meta-analysis reported that individuals with IBD had significantly higher odds of VDD compared with controls [65]. According to Endocrine Society guidelines, serum 25(OH)D_3_ levels below 20 ng/mL (50 nmol/L) are classified as deficient, whereas levels between 21–29 ng/mL (52.5–72.5 nmol/L) indicate an insufficiency [66]. In patients with IBD, VDD results from impaired intestinal formation and absorption of vitamin D micelles and chylomicrons, as well as insufficient UVB exposure, which may be influenced by factors such as outdoor activity, latitude, skin pigmentation, and reduced time spent outdoors due to disease-related limitations [7,67]. Additional risk factors include insufficient dietary intake, smoking, and corticosteroid therapy [68].

Reduced vitamin D concentrations have been associated with increased disease activity, mucosal inflammation, higher relapse risk, and poorer quality of life, as reflected by increased Crohn’s Disease Activity Index (CDAI) and partial Mayo scores and elevated C-reactive protein (CRP) levels. Therefore, VDD may represent both a contributing factor to disease progression and a consequence of the inflammatory process in IBD [6,16]. Multiple retrospective and observational studies have linked VDD to active IBD and its complications, including hospitalization, surgery, and malignancy, suggesting its potential use as a biomarker of disease activity. Vitamin D levels have also been proposed for monitoring patients receiving infliximab therapy due to their correlation with drug concentrations. Prospective data indicate that VDD appears after disease onset and treatment in CD, supporting the hypothesis that it is a consequence rather than a cause of the disease [69]. Severe 25(OH)D_3_ deficiency was significantly associated with ileocolonic involvement and complicated disease behavior in CD, and with greater disease extent in UC. Furthermore, severe deficiency represented an independent risk factor for intestinal surgery during follow-up in both CD and UC [16]. A cross-sectional study comparing 25(OH)D_3_ levels in patients with IBD and irritable bowel syndrome (IBS) found that VDD was particularly common among patients with CD. Lower serum 25(OH)D_3_ levels were associated with increased inflammatory markers, including CRP and fecal calprotectin (FC), suggesting a relationship between VDD and disease activity in CD. Stratification according to inflammatory status, defined by FC levels above or below 100 µg/g stool, further showed that patients with higher FC values had lower 25(OH)D_3_ concentrations, supporting an inverse association between vitamin D status and intestinal inflammation [70].

### 4.3. Vitamin D and Susceptibility to Intestinal Inflammation in IBD

Vitamin D is essential for intestinal homeostasis and may influence IBD onset, relapse, and progression through its effects on mucosal barrier integrity, immune responses, and gut microbiota composition. Preclinical data show that vitamin D/VDR signaling supports tight and adherens junctions, thereby maintaining barrier integrity, and may also indirectly affect mucus production [71]. Vitamin D additionally modulates the expression of zonulin, a regulator of tight junction disassembly. Increased zonulin levels, commonly observed in IBD, contribute to barrier dysfunction, whereas vitamin D suppresses its release, thereby enhancing epithelial integrity. In addition, vitamin D modulates the immune system by inhibiting pro-inflammatory Th1 and Th17 cells while promoting Treg development, thereby supporting immune tolerance [72]. It suppresses the production of Th1-associated pro-inflammatory cytokines, including IFN-γ, IL-12, and TNF-α, which are critically involved in the pathogenesis of IBD. Conversely, vitamin D may promote the Th2-associated responses, characterized by anti-inflammatory cytokines such as IL-4, IL-5, and IL-13, and suppress IL-17 production by Th17 [5]. Although CD is largely Th1/Th17-driven and UC involves Th2 and innate pathways, vitamin D appears to influence both conditions [72]. Unlike Th1 cells, whose pathogenic role in IBD is well established, the function of Th17 cells remains debated, as both protective and pathogenic effects have been reported. Th17 cells support mucosal integrity by enhancing antimicrobial peptide production and promoting epithelial repair; however, they may also contribute to inflammation through pro-inflammatory cytokine secretion, disruption of Th17/Treg balance, and involvement in mucosal damage [73].

### 4.4. VDR Signaling in Macrophage Activation and Polarization in IBD

**Figure 2:**
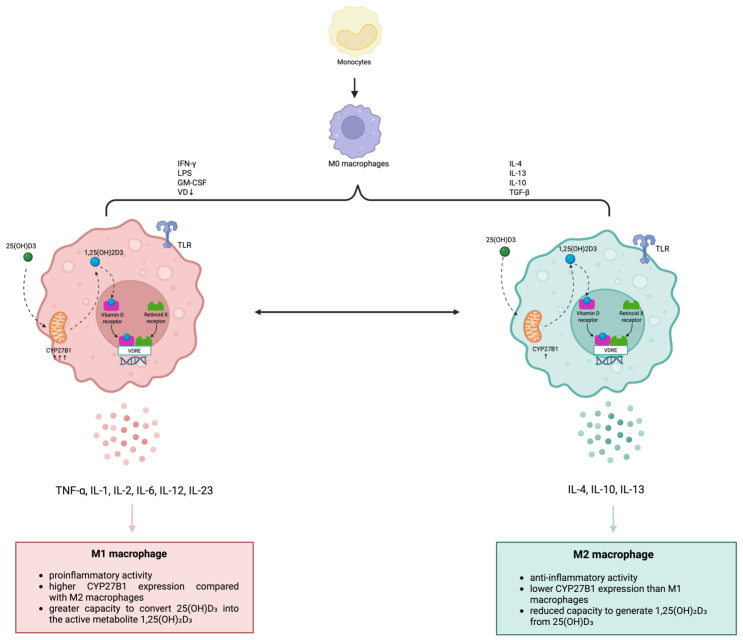
Macrophage polarization: M1 and M2 subtypes. Monocytes differentiate into naive M0 macrophages, which can polarize into M1 or M2 phenotypes depending on the microenvironmental stimuli. M1 polarization is induced by signals such as IFN-γ, LPS (Lipopolysaccharide), and GM-CSF (Granulocyte–Macrophage Colony-Stimulating Factor), and is associated with increased expression of CYP27B1, leading to an enhanced conversion of 25(OH)D_3_ into the active metabolite 1,25(OH)_2_D_3_. This active form binds to the vitamin D receptor (VDR), forming a heterodimer with the retinoid X receptor (RXR), which regulates gene transcription via vitamin D response elements (VDRE). M1 macrophages exhibit a pro-inflammatory profile characterized by the secretion of cytokines such as TNF-α, IL-1, IL-2, IL-6, IL-12, and IL-23. In contrast, M2 polarization is driven by cytokines such as IL-4, IL-13, IL-10, and TGF-β and display lower CYP27B1 expression, having a reduced capacity to generate 1,25(OH)_2_D_3_. Created in BioRender. Grigoraș, A. (2026) https://BioRender.com/org4kk4 (accessed on 1 May 2026).

Monocytes and macrophages are key contributors to host defense against infections through their production of pro-inflammatory cytokines. Microbial components derived from bacteria, viruses, and fungi are detected by Toll-like receptors (TLRs) expressed on the surface of these cells, leading to enhanced expression of the enzyme CYP27B1 and the VDR. As mentions before, CYP27B1 is responsible for the conversion 25(OH)D_3_ into its active form, 1,25(OH)_2_D_3_, therefore upregulating the expression of antimicrobial peptides, including cathelicidin and β-defensin 2, enhancing host defense against infections [74]. M1 cells exhibiting higher CYP27B1 expression and a greater capacity to convert 25(OH)D_3_ to active 1,25(OH)_2_D_3_ than M2 cells [8]. A study comparing macrophage function in patients with CD and healthy controls showed that CD macrophages were functionally similar to those of controls, but active vitamin D significantly reduced pro-inflammatory cytokine production, particularly in M1 macrophages. These findings support the anti-inflammatory role of vitamin D in macrophage regulation and suggest that dysregulation of the M1/M2 balance, influenced by cytokines, microbial products, and vitamin D signaling, may contribute to IBD pathogenesis (Figure 2) [42].

**Figure 3:**
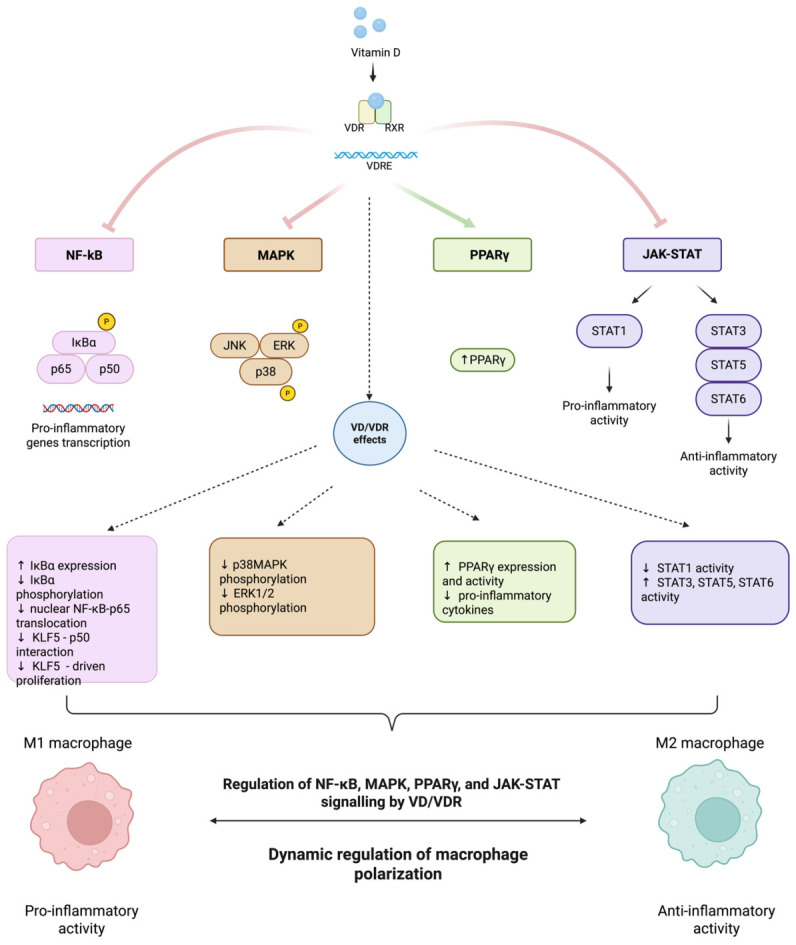
Vitamin D/VDR-mediated signaling pathways involved in macrophage polarization. Vitamin D regulates macrophage polarization through coordinated modulation of several intracellular signaling pathways involved in inflammatory and immune responses, including nuclear factor-kappa B (NF-κB), mitogen-activated protein kinase (MAPK), peroxisome proliferator-activated receptor gamma (PPARγ), and Janus kinase/signal transducer and activator of transcription (JAK/STAT) signaling. Following binding of the active vitamin D, 1,25(OH)_2_D_3_, to the vitamin D receptor (VDR), downstream signaling mechanisms suppress pro-inflammatory pathways associated with M1 macrophage activation while promoting anti-inflammatory responses characteristic of the M2 phenotype. Vitamin D inhibits NF-κB activation by increasing inhibitor of nuclear factor kappa B alpha (IκBα) stability and reducing NF-κB nuclear translocation, thereby decreasing the expression of pro-inflammatory mediators. In parallel, vitamin D attenuates MAPK signaling through inhibition of p38 MAPK and extracellular signal-regulated kinase 1/2 (ERK1/2) phosphorylation, limiting inflammatory cytokine production and M1-associated macrophage responses. In addition, vitamin D promotes macrophage reprogramming toward an anti-inflammatory phenotype through activation of PPARγ signaling, which enhances the expression of M2-associated mediators, while suppressing pro-inflammatory cytokines. Regulation of the JAK/STAT pathway further contributes to these effects, as vitamin D suppresses STAT1-mediated inflammatory signaling and enhances STAT3, STAT5, STAT6 activation, promoting M2 polarization and immune homeostasis. Collectively, these interconnected mechanisms highlight the central immunomodulatory role of vitamin D in macrophage plasticity and the resolution of inflammation. Created in BioRender. Grigoraș, A. (2026) https://BioRender.com/3kf2gm4 (accessed on 1 May 2026).

Vitamin D modulates macrophage polarization through multiple cellular signaling mechanisms, primarily by regulating the nuclear factor-kappa B (NF-κB), mitogen-activated protein kinase (MAPK), vitamin D receptor–peroxisome proliferator-activated receptor gamma (VDR–PPARγ), and Janus kinase–signal transducer and activator of transcription (JAK/STAT) pathways (Figure 3) [75,76].

#### 4.4.1. Vitamin D/VDR–NF-κB Signaling in Macrophage Polarization

Vitamin D exerts significant anti-inflammatory effects through inhibition of the NF-κB signaling pathway, as shown in a study conducted on murine macrophages. Specifically, vitamin D reduces TNF-α secretion and suppresses NF-κB activity by decreasing nuclear NF-κB p65 levels while promoting its cytosolic retention. This regulatory effect is mediated by increased inhibitor of nuclear factor kappa B alpha (IκBα) expression, resulting from enhanced mRNA stability and reduced IκBα phosphorylation, which collectively inhibit NF-κB nuclear translocation and downstream inflammatory signaling [77,78]. In addition to its interaction with the NF-κB p65 subunit, vitamin D binds to the NF-κB p50 subunit and modulates downstream signaling. Lipopolysaccharide (LPS) promotes macrophage proliferation via Krüppel-like factor 5 (KLF5) upregulation and its interaction with NF-κB p50, leading to activation of cell cycle genes. In contrast, vitamin D enhances VDR expression and VDR p50 binding, which competitively disrupts KLF5 p50 interaction, thereby suppressing KLF5-driven proliferative signaling and attenuating macrophage proliferation [79]. In IBD, modulation of NF-κB activity influences the balance between pro-inflammatory and anti-inflammatory macrophage phenotypes. Accordingly, increased expression of nicotinamide phosphoribosyltransferase (NAMPT) has been associated with disease severity, while its inhibition attenuates inflammation by suppressing NF-κB signaling and promoting macrophage polarization toward the anti-inflammatory M2 phenotype. Similarly, corticosteroids exert part of their therapeutic effect in IBD through NF-κB inhibition and the induction of M2 macrophage polarization [76].

The interferon regulatory factor (IRF) family plays a central role in the regulation of immune cell function and macrophage polarization. In particular, IRF5 promotes a pro-inflammatory M1 phenotype by inducing the expression of cytokines such as TNF-α, IL-12, and IL-23, while simultaneously repressing IL-10 transcription. In contrast, IRF4 is associated with the anti-inflammatory M2 phenotype and drives the expression of M2-specific markers. In addition, IRF4 and IRF5 competitively bind to myeloid differentiation primary response 88 (MYD88), and the balance between these transcription factors is essential for the regulation of macrophage polarization. NF-κB signaling is closely interconnected with IRF pathways, as IκB kinase activates both NF-κB and IRF5. In this context, vitamin D modulates macrophage responses by enhancing VDR interaction with NF-κB p50, suppressing pro-inflammatory signaling, and promoting an anti-inflammatory phenotype through increased IRF4 expression and reduced IRF5 activation [80].

#### 4.4.2. Vitamin D/VDR–MAPK Signaling in Macrophage Polarization

The MAPK signaling pathway, which includes the c-Jun N-terminal kinase (JNK), extracellular signal-regulated kinase (ERK), and p38 MAPK subfamilies, plays an important role in the regulation of inflammation and macrophage polarization in IBD. Experimental studies in dextran sodium sulfate (DSS)-induced colitis have demonstrated that activation of the ERK and p38 MAPK pathways is associated with intestinal inflammation, apoptosis, and increased abundance of pro-inflammatory M1 macrophages [76]. Vitamin D has been shown to exert potent anti-inflammatory effects by inhibiting MAPK signaling pathways in immune cells, particularly macrophages. Specifically, it reduces the activation of key MAPK components, including phosphorylated p38 MAPK and ERK1/2, which are critical mediators of pro-inflammatory signaling. Furthermore, it reduces inflammatory responses induced by LPS and pro-inflammatory cytokines, such as IL-6 and IL-1β. These findings suggest that vitamin D negatively regulates MAPK-dependent inflammatory pathways, consequently limiting macrophage activation and polarization toward the pro-inflammatory M1 phenotype [75].

#### 4.4.3. Vitamin D/VDR–PPARγ Signaling in Macrophage Polarization

PPARγ also represents a key regulator of macrophage polarization, promoting the anti-inflammatory M2 phenotype while inhibiting polarization toward the pro-inflammatory M1 phenotype. Its expression is influenced by cytokines, metabolic pathways, and signaling axes such as mechanistic target rapamycin–semephorin 6D–peroxisome proliferator-activated receptor gamma (mTOR–Sema6D–PPARγ), highlighting the close relationship between cellular metabolism and macrophage function. In M1 macrophages, inflammatory stimuli such as LPS and IFN-γ promote aerobic glycolysis, whereas IL-4-mediated lipid metabolism in M2 macrophages upregulates PPARγ expression. Therefore, modulation of PPARγ signaling represents an important mechanism for regulating macrophage polarization and controlling inflammatory responses [81].

Vitamin D has been identified as an important regulator of macrophage polarization, promoting a shift from the pro-inflammatory M1 phenotype toward the anti-inflammatory M2 phenotype. Experimental evidence indicates that vitamin D reduces the production of pro-inflammatory cytokines such as TNF and IL-12 while increasing IL-10 levels in M1 macrophages, effects that are dependent on the VDR–PPARγ signaling axis. Mechanistically, modulation of VDR expression leads to enhanced PPARγ activity, which drives macrophage reprogramming toward an anti-inflammatory state and is associated with reduced inflammation. Collectively, these findings highlight the role of vitamin D in regulating macrophage function through VDR–PPARγ-dependent pathways, contributing to the attenuation of inflammatory responses [75].

Both VDR and PPARγ, members of the nuclear receptor superfamily, play important roles in regulating macrophage polarization. Upon activation by specific ligands, PPARγ promotes macrophage polarization toward the anti-inflammatory M2 phenotype. Similarly, VDR, activated by 1,25(OH)_2_D_3_, functions as a transcription factor that regulates macrophage activity in a manner comparable to PPARγ. The VDR and PPARγ pathways are interconnected, influencing key cellular processes such as growth, differentiation, and immune responses, although the precise mechanisms underlying their interaction remain unclear [82]. 1,25(OH)_2_D_3_ suppresses the LPS-induced production of pro-inflammatory mediators, including TNF-α, IL-1β, and cyclooxygenase-2 (COX-2), while enhancing the secretion of the anti-inflammatory cytokine IL-10 in macrophages. Deletion of VDR in macrophages exacerbates colitis and is associated with elevated expression of pro-inflammatory cytokines in the inflamed colon, indicating that vitamin D signaling in innate immune cells plays a critical role in regulating intestinal inflammation [80].

#### 4.4.4. Vitamin D/VDR–JAK/STAT Signaling in Macrophage Polarization

The JAK/STAT signaling pathway plays a crucial role in the regulation of macrophage polarization and inflammatory responses in IBD. Activation of STAT1 is primarily associated with pro-inflammatory M1 macrophage polarization, whereas STAT3, STAT5, and STAT6 promote the anti-inflammatory M2 phenotype. Experimental and therapeutic studies conducted both in vitro using cell culture models (LPS/IFN-γ-stimulated M1 RAW264.7 macrophages) and in vivo in murine systems (male C57BL/6 mice), have demonstrated that modulation of JAK/STAT signaling can influence disease progression by regulating macrophage phenotype and immune balance. In particular, inhibition of STAT1 signaling reduces M1-associated inflammatory responses, while activation of STAT5 and STAT6 pathways promotes M2 polarization, intestinal repair, and anti-inflammatory activity [76,83]. Vitamin D promotes anti-inflammatory macrophage polarization through coordinated activation of STAT6 and VDR–PPARγ signaling pathways. Experimental evidence indicates that vitamin D enhances STAT6 activation, leading to increased IL-4 and IL-10 expression, elevated arginase activity, and a shift toward an M2 macrophage phenotype. This effect is accompanied by a reduction in pro-inflammatory cytokines, including TNF, IL-6, and IL-12, while promoting anti-inflammatory mediators such as IL-10 and TGF-β. Collectively, these findings demonstrate that vitamin D acts as a key immunomodulatory factor that drives macrophage polarization toward an anti-inflammatory phenotype through STAT6- and VDR–PPARγ-dependent mechanisms [75].

### 4.5. VDR-Mediated Regulation of Macrophage–IL-23 Production in IBD

VDR plays a pivotal role in modulating innate and adaptive immunity and is expressed in a wide range of immune cells, including lymphocytes, monocytes, macrophages, and dendritic cells (DCs). By regulating cytokine production in antigen-presenting cells, as well as T and B lymphocytes, VDR contributes to immune system homeostasis, with 1,25(OH)_2_D_3_ serving as a key immunomodulatory ligand. VDR also functions as a transcription factor that modulates macrophage activity, promoting the shift toward the anti-inflammatory M2 phenotype. This regulatory role is analogous to that of PPARγ, which also drives M2 polarization upon ligand activation [82]. Previous immunotherapeutic strategies in IBD have focused on modulating macrophage polarization by suppressing the pro-inflammatory M1 subset and promoting the anti-inflammatory M2 phenotype, thereby reducing intestinal tissue damage mediated by M1-derived cytokines and other inflammatory mediators [76].

The ability of VDR to promote M2 macrophage polarization is particularly important in IBD, as M1 macrophages are a major source of IL-23 in response to bacterial and fungal stimuli. A study using transcriptomic analysis and single-cell RNA sequencing of mononuclear phagocytes and peripheral blood mononuclear cells from healthy individuals and patients with IBD identified specific monocyte subsets responsible for IL-23 expression. IL-1α, IL-1β, and IL-10 were shown to regulate IL-23-producing monocytes, while an inflammatory monocyte transcriptional signature was associated with active disease and resistance to anti-TNF therapy. These findings support the role of IL-23-producing monocytes in chronic intestinal inflammation and highlight their potential as therapeutic targets. IL-23 further promotes Th17- and Th1-associated immune responses, thereby driving intestinal inflammation. Accordingly, the IL-23/Th1/Th17 axis represents a central pathway in the regulation of immune responses and the maintenance of chronic gut inflammation [76,84]. IL-23 belongs to the IL-12 family and has been implicated in the pathogenesis of several inflammatory disorders, including IBD, psoriasis and psoriatic arthritis [85]. Together with IL-12, IL-23 contributes to pro-inflammatory immune signaling, and the overlap between these pathways is reflected by their shared p40 cytokine subunit and IL-12Rβ1 receptor subunit. For this reason, therapeutic targeting of IL-12/IL-23 has emerged as a promising strategy for the treatment of IBD [86].

## 5. Therapeutic Strategies Targeting the VDR–Macrophage–IL-23 Axis in IBD

### 5.1. Current Therapeutic Strategies in IBD

Multiple therapeutic options are currently available for the management of IBD. Nevertheless, a significant proportion of patients experience inadequate response or treatment discontinuation due to adverse events. Aminosalicylates (5-ASA) are the standard first-line therapy for uncomplicated UC. Patients with inadequate response require systemic corticosteroids or, in mild-to-moderate cases, Budesonide. In steroid-refractory or steroid-dependent cases, treatment escalation to biologic agents, calcineurin inhibitors, JAK inhibitors, or Sphingosine-1-Phosphate (S1P) receptor modulators are indicated. Unlike UC, 5-ASA agents lack demonstrated efficacy in CD and are not recommended for induction or maintenance therapy. Budesonide is indicated for mild ileocecal or right-sided disease, whereas systemic corticosteroids are required in cases of severe activity or extensive small bowel involvement. Steroid-refractory or steroid-dependent disease necessitates escalation to biologic therapies, including anti-TNF agents (infliximab, adalimumab), monoclonal antibodies (ustekinumab), and anti-integrin therapy (vedolizumab), with newer agents such as JAK inhibitors (upadacitinib) and selective IL-23p19 monoclonal antibodies (risankizumab) emerging as additional options, particularly in patients with prior biologic failure [10,11]. In addition, adjunctive therapies, such as vitamin D supplementation in patients with VDD, may complement standard IBD therapy, although current evidence remains insufficient [12].

### 5.2. Vitamin D and VDR-Based Therapeutic Strategies

Vitamin D exerts multiple roles beyond calcium homeostasis and bone metabolism, being involved in immune regulation and potential gut microbiota modulation [87]. Because VDD is associated with IBD, supplementation with vitamin D in patients with IBD has gained increasing interest in recent years [65,88]. In a randomized, double-blind placebo-controlled trial in CD patients in remission, oral vitamin D_3_ treatment with 1200 IU/day significantly increased serum 25(OH)D_3_ levels. Although the relapse rate was lower in patients receiving vitamin D compared with those receiving placebo, this difference did not reach statistical significance [89].

More recently, a meta-analysis reported that vitamin D supplementation may reduce the risk of clinical relapse in patients with IBD, with the most consistent effect observed in CD patients. However, subgroup analyses, particularly for UC patients, did not reach statistical significance, due to limited number of included studies and small sample sizes [90]. In another study, vitamin D supplementation was associated with reduced IBD-related healthcare utilization, including fewer emergency department visits, hospitalizations, and corticosteroid use, supporting the potential value of vitamin D as an adjunct to standard therapy [91].

Although VDD is associated with IBD, most available data derive from observational studies; therefore, a causal relationship cannot be established [65]. In addition, it is unclear whether VDD is a cause or a consequence of IBD, as the vitamin D status in IBD may be influenced by multiple confounding factors, such as impaired nutrient absorption, restrictive dietary intake, reduced sunlight exposure during immunosuppressive therapy, corticosteroid use, previously intestinal surgery, and disease activity [92]. Randomized studies are still heterogeneous in terms of the optimal dosing strategies and the targeted serum levels for sustaining long-term benefits. Therefore, oral vitamin D supplementation may represent a safe and inexpensive adjunctive therapy, but at present, it should not be regarded as an independent disease-modifying therapy in IBD [15,91].

Apart from vitamin D supplementation, some studies have investigated the potential therapeutic effects of VDR agonist on patients with IBD. For example, 1α,25(OH)_2_-19-nor-14,20-bisepi-23-yne-vitamin D_3_ (TX527) inhibited cell proliferation and TNF-α production in peripheral blood mononuclear cells of CD patients [93,94]. Laverny et al. showed that intrarectal administration of 1α,25(OH)_2_-16-ene-20-cyclopropyl-vitamin D_3_ (BXL-62), another potent VDR agonist, induced recovery from clinical symptoms of colitis in experimental mice IBD, at normocalcemic doses [94]. In another study, paricalcitol, a vitamin D analog that activates VDR, significantly ameliorated the disease severity in 2,4,6-trinitrobenzene sulfonic acid (TNBS)-induced colitis, a model characterized by Th1-mediated intestinal inflammation [95]. However, these findings remain mainly preclinical, and further clinical studies are required to determine whether VDR agonists are comparable or superior to vitamin D supplementation, or whether their effects may be complementary.

### 5.3. Direct IL-23-Targeting Strategies

The most clinically advanced therapeutic strategies are derived from direct inhibition of IL-23. These strategies can be divided into selective and non-selective approaches based on their molecular targets. Selective inhibitors, such as risankizumab, mirikizumab guselkumab, specifically neutralize the p19 subunit of IL-23, thereby modulating the IL-23/Th17 axis without affecting IL-12 signaling. In contrast, non-selective inhibitors, such as ustekinumab, which target the shared p40 subunit, block both IL-23 and IL-12, exerting broader immunomodulatory effects (Table 1) [96].

Ustekinumab, a monoclonal antibody targeting the p40 subunit shared by IL-12 and IL-23, suppresses both Th1 and Th17 mediated inflammatory pathways. Clinical trials have demonstrated its efficacy in both CD and UC, with significantly higher remission rates compared with placebo [97]. Ustekinumab has demonstrated efficacy in both the induction and maintenance of clinical remission in patients with active CD and UC [101]. Through dual inhibition of IL-12 and IL-23, ustekinumab exerts broad immunomodulatory effects while maintaining aspects of host defense [97,102]. However, only patients who responded to ustekinumab induction were re-randomized, limiting conclusions for primary non-responders; therefore, the maintenance data should be interpreted cautiously. In addition, reported deaths and cancer cases during follow-up, the lack of direct head-to-head comparison with other biologic treatments, and concerns regarding the cost-effectiveness represent important limitations [103,104].

Risankizumab is a humanized monoclonal antibody that selectively targets the p19 subunit of IL-23. Through inhibition of this subunit, it suppresses downstream cytokine signaling and consequently attenuates the IL-23-mediated inflammatory cascade [98]. In a phase 3b head-to-head clinical trial involving patients with moderate-to-severe CD who previously had unacceptable side effects or an inadequate response to anti-TNF therapy, risankizumab was noninferior to ustekinumab for clinical remission at week 24 and superior for endoscopic remission at week 48 [103]. These findings suggest that selective IL-23p19 inhibition may provide greater benefits for patients with moderate-to-severe CD, while also being effective and well tolerated in this population [103,105]. However, the 180 mg dose did not significantly improve stool frequency and abdominal pain score clinical remission, and common adverse events included worsening CD, arthralgia, and headache [106].

Mirikizumab, another selective IL-23p19 inhibitor, has demonstrated efficacy in both UC and CD. In UC, evidence from Phase III LUCENT-1 and LICENT-2 trials showed that mirikizumab significantly improved clinical remission rates compared with placebo during both induction and maintenance phases [107]. In CD, earlier Phase II data showed that mirikizumab induced endoscopic and clinical responses and demonstrated durable efficacy through week 52 [108]. More recently, the Phase III VIVID-1 trial confirmed its efficacy in patients with moderate-to-severe active CD, showing significantly higher rates of clinical remission and endoscopic response compared with placebo [109]. Nevertheless, current evidence remains limited by the lack of long-term real-world data, and further studies are needed to assess durability of response, loss of response over time, and safety in broader IBD populations [107,109,110].

Guselkumab has also been evaluated in phase 3 double-blind, randomized, placebo-controlled induction and maintenance studies (the QUASAR program), where it was found to be effective and safe as an induction and maintenance therapy in patients with moderate-to-severe active UC [111]. However, these findings have similar limitations, especially the need for more long-term clinical practice data [112].

### 5.4. Indirect Modulation of IL-23 Signaling

JAK inhibitors represent a promising therapeutic approach in IBD, having demonstrated clinical efficacy in multiple trials. Unlike monoclonal antibodies, which selectively target individual cytokines, JAK inhibitors modulate multiple cytokine-dependent signaling pathways, potentially enhancing therapeutic responses in certain patient subgroups [113]. JAK family, including JAK1, JAK2, JAK3, and tyrosine kinase 2 (TYK2), forms an integral component of transmembrane cytokine receptor complexes. Ligand binding to these receptors triggers JAK recruitment, phosphorylation, and subsequent activation of STATs [114]. The JAK/STAT pathway mediates signaling downstream of both IL-12 and IL-23 receptors, which are primarily coupled to JAK2 and TYK2. Ligand binding induces receptor conformational changes that facilitate assembly of the receptor complex and subsequent activation of JAK2 and TYK2, thereby initiating intracellular signaling cascades [3]. The JAK/STAT signaling pathway is critically involved in both innate and adaptive immunity, and regulates key cellular processes including proliferation, survival, differentiation, and migration [114].

Currently, two JAK inhibitors are approved by the FDA for the treatment of IBD, tofacitinib and upadacitinib, while filgotinib has received approval in the European Union, Great Britain, and Japan. Tofacitinib functions as a pan-JAK inhibitor, exhibiting high affinity for JAK1 and JAK3, with additional activity against JAK2 at higher doses, and is currently approved in the United States exclusively for UC. In contrast, upadacitinib and filgotinib are selective JAK1 inhibitors; upadacitinib is indicated for both moderate-to-severe UC and CD, whereas filgotinib is approved for moderate-to-severe UC in Europe, Great Britain, and Japan (Table 1) [100,115].

Unlike biologic therapies, JAK inhibitors have a short half-life, allowing interference with the immunosuppressive effect in case of infection or pregnancy. They are efficient at lower doses and in contrast with biologic therapies, which typically neutralize a single extracellular cytokine, JAK inhibitors modulate intracellular signaling pathways [116]. Nevertheless, this broader intracellular mechanism is also associated with concerning complications, including heart-related events, blood clots, and death. These risks have been initially signaled for tofacitinib administration, but the warnings have also been extended to other JAK inhibitors, including upadacitinib [117].

Overall, these therapeutic strategies differ in their clinical advantages and limitations. While IL-23-targeting therapies offer more selective cytokine inhibition and favorable efficacy in specific IBD populations, JAK inhibitors provide broader intracellular modulation and potential benefits in selected subgroups but require careful risk assessment.

## 6. Limitations

This review has several limitations that should be acknowledged. First, as a narrative review, it may be affected by publication bias, since studies reporting positive associations between vitamin D, VDR signaling, macrophage polarization, IL-23 activity, and IBD may be more likely to be published than studies reporting negative or inconclusive results. Second, the included evidence is heterogeneous, comprising in vitro studies, animal models, observational human studies, randomized clinical trial, and therapeutic trial, each with distinct design characteristics.

Another important limitation is that much of the existing evidence linking VDR signaling to macrophage polarization and IL-23 regulation is derived mainly from preclinical models, while direct confirmation in human intestinal mucosa remains limited. In addition, although VDD is frequently associated with IBD, most available clinical findings come from observational studies; therefore, causality cannot be established. Regarding vitamin D supplementation, studies remain heterogeneous in terms of optimal dosing strategies and the target serum 25(OH)D_3_ levels required to maintain long-term benefits. Finally, evidence regarding the combined modulation of vitamin D/VDR pathways and IL-23-targeted therapies remains scarce, and the influence of factors as VDR gene polymorphisms, microbiota composition, prior biologic exposure, and long-term treatment response remains insufficiently defined.

## 7. Conclusions and Future Directions

IBD is a complex, immune-mediated disorder driven by dysregulated interactions between genetic susceptibility, environmental triggers, microbial factors, and aberrant mucosal immune responses. The interplay between vitamin D, VDR signaling, macrophage polarization, and IL-23-driven inflammation represents a compelling and increasingly relevant immunoregulatory axis in IBD immunopathogenesis. IL-23 links innate and adaptive immunity by promoting Th17 responses, while macrophages contribute to this network as both sources of IL-23 and regulators of mucosal immune homeostasis. VDR signaling may modulate this axis by limiting pro-inflammatory macrophage activation, reducing IL-23 production, supporting immune tolerance, and reinforcing epithelial barrier integrity.

Future research should focus on large-scale, randomized clinical trials to determine whether vitamin D supplementation or selective VDR agonists can improve disease activity, relapse prevention, mucosal healing, and response to IL-23-targeted therapies. These studies should also explore the impact of VDR gene polymorphisms on treatment effect, and stratify patients by baseline vitamin D status, IBD subtype, and prior biologic exposure. Additionally, investigations into the interactions between vitamin D, gut microbiota, host genetics, and IL-23-mediated inflammation could inform precision medicine approaches, optimize patient stratification, and guide preventive strategies. Collectively, these efforts may establish vitamin D not only as a supportive adjunct but also as a modifiable factor integrated with emerging IL-23-targeted therapies for the personalized management of IBD.

## Figures and Tables

**Table 1:** Therapeutic modulation of Interleukin (IL)-23 in inflammatory bowel disease.

Strategy	Therapeutic Class	Representative Agents	Mechanism of Action	References
Direct IL-23 targeting (non-selective IL-12/IL-23 blockade)	Monoclonal antibodies (anti-p40)	Ustekinumab	Binds the shared p40 subunit of IL-12 and IL-23, inhibiting signaling through both cytokine pathways	[96,97]
Direct IL-23 targeting (selective IL-23 blockade)	Monoclonal antibodies (anti-p19)	Risankizumab Mirikizumab Guselkumab	Selectively binds the p19 subunit of IL-23, preserving IL-12 signaling and suppressing IL-23-driven inflammation	[96,98,99]
Indirect IL-23 pathway modulation	JAK inhibitors	Tofacitinib Upadacitinib Filgotinib	Inhibit JAK–STAT signaling, interfering with cytokine pathways involved in IL-23-mediated immune responses	[100]

## Data Availability

No new data were created or analyzed in this study.
